# Freshly frozen E18 rat cortical cells can generate functional neural networks after standard cryopreservation and thawing procedures

**DOI:** 10.1007/s10616-014-9700-9

**Published:** 2014-02-23

**Authors:** Kim Quasthoff, Stefano Ferrea, Wiebke Fleischer, Stephan Theiss, Alfons Schnitzler, Marcel Dihné, Janine Walter

**Affiliations:** 1 Department of Neurology, Medical Faculty, Heinrich-Heine University, Moorenstr. 5, 40225 Düsseldorf, Germany; 2 Department of Neurology and Epileptology, Hertie Institute for Clinical Brain Research, Eberhard-Karls-University, Hoppe-Seyler-Straße 3, 72076 Tübingen, Germany; 3 Medical Faculty, Institute of Clinical Neuroscience and Medical Psychology, Heinrich-Heine University, Universitätsstr. 1, 40225 Düsseldorf, Germany

**Keywords:** Primary rat E18 cortical neurons, Cryopreservation of cells, In vitro-neuronal network activity (ivNNA)

## Abstract

Primary dissociated brain tissue from rodents is widely used in a variety of different scientific methods to investigate cellular processes in vitro. Often, for this purpose cell cultures need to be generated just on time, requiring extensive animal lab infrastructure. We show here that cryopreservation and thawing of dissociated tissue from rat cerebral cortex at embryonic day 18 is feasible without affecting its ability to form functional neuronal networks in vitro. Vitality of fresh and re-thawed cortical cells was comparable, assessed by CellTiter-Blue-assay, CytoTox-ONE assay, immunocytochemical characterization and in vitro neuronal network activity recordings on microelectrode arrays. These findings suggest that planning and execution of experiments might be considerably facilitated by using cryo-preserved neurons instead of acutely dissociated neural cultures due to fewer logistical issues with regard to animal breeding and pregnancy timed preparations.

## Introduction

In neuroscience fields, usage of embryonic brain tissue and isolation and cultivation of neural cells from specific brain regions is a very common technique (Giordano and Costa 2011; Negishi et al. 2002, 2003). A limiting factor in such experiments is the number of pregnant animals available, their cost, and the time required to reach specific developmental ages during pregnancy. Also, processing brain tissue of numerous embryos synchronously at specific days needs rigorous planning, expertise and manpower. In recent years, it has also been possible to purchase specifically cryo-preserved neurons from different companies. However, this approach is considerably limited if mutant genetic backgrounds are investigated. Recently, cryo-preservation of different brain cell and tissue types from various species has been described. For instance, human neuronal cells and cells from bovine brains were successfully cryo-preserved (Ballarin and Peruffo 2012; Mattson and Rychlik 1990; Silani et al. 1988; Taupin 2009), as well as rat cortical brain tissue blocks (Milosevic et al. 2005; Petite and Calvet 1995; Rahman et al. 2010), and human neural stem and progenitor cells (Silani et al. 1988). The developmental stage in which the mentioned cultures were frozen varies from very early embryonic to fetal developmental stages, but none of the above mentioned studies used electrophysiological methods to prove the vitality of the cryo-preserved cells after thawing.

We show here that dissociated primary rat E18 cortical cells can be frozen and thawed by standard laboratory procedures without severely reducing their viability or capability to generate in vitro-neuronal network activity which was assessed by using microelectrode arrays (MEAs). MEAs detect electrophysiological network activity like action potentials and synchronous neuronal network (population bursts) activity via multiple extracellular electrodes (Berdondini et al. 2009; Chiappalone et al. 2006; Grumet et al. 2000; Heuschkel et al. 2002; Ivenshitz and Segal 2010; Jolly et al. 1997; Novellino et al. 2011; Otto et al. 2003; Reppel et al. 2004; Schock et al. 2012).

## Materials and methods

### Cell culture and preparation

All animal experiments performed were approved by the animal care committees of the Heinrich-Heine-University of Düsseldorf and the state of North Rhine-Westphalia, Germany. Primary cortical cells were derived from Wistar rats at embryonic day 18. Briefly, embryonic day 18 pregnant Wistar rats were euthanized by Isoflurane and cervically dislocated. Embryos were dissected and their brains isolated. Cortices were separated and exempt from meninges. Afterwards, they were chopped with a scalpel and collected in ice cold low glucose DMEM medium (Invitrogen, Karlsruhe, Germany). The medium was centrifuged for 1 min at 2,000 rpm and the supernatant discarded. In order to produce a single cell suspension, cortices from 3 embryos were digested with 0.05 % trypsin (Invitrogen, Karlsruhe, Germany) for 10 min at 37 °C. Digestion was stopped by adding 10 ml of low glucose DMEM complemented with 10 % fetal calf serum (FCS) (Fisher Scientific, Schwerte, Germany). Cell suspension was centrifuged for 1 min at 2,000 rpm, the supernatant was discarded, and the cells were gently resuspended in 2 ml low glucose DMEM. The cell suspension was filled up with medium and centrifuged again for 5 min at 1,500 rpm. Thereafter, supernatant was discarded and cells were resuspended in supplemented DMEM/F12 N2 medium containing 5 % FCS. After counting, the cells were seeded or frozen, respectively. Directly after the dissection, 1.5 × 10^5^ cells were plated on poly-d-lysine- and laminin-coated MEAs for neurophysiological measurements, another part was counted and frozen at −80 °C and a third part was plated on cell culture plates for viability assays and immunocytochemistry. The fresh cells were incubated in D-MEM F12 N2/FCS medium for the first 2.5 days supplemented with Glutamax (Invitrogen, Karlsruhe, Germany) and Penicillin/Streptomycin (Invitrogen, Karlsruhe, Germany). Afterwards, a complete medium change to B27 supplemented Neurobasal medium with Glutamax (Invitrogen, Karlsruhe, Germany) and Penicillin/Streptomycin (Invitrogen, Karlsruhe, Germany) was performed. Cells were kept in a humidified atmosphere (5 % CO_2_/95 % air) at 37 °C. Medium was replaced twice a week, with a half medium exchange. The frozen cells were left at −80 °C for 3 days and then thawed. After counting, the cells were plated on poly-d-lysine- and laminin-coated MEAs (Biopur, Reinach, Switzerland). From this step on, they underwent the same treatment as the fresh cells. For the cryopreservation we used a cryo-medium consisting of FCS and 20 % DMSO (Sigma-Aldrich, Hamburg, Germany), freshly prepared on the day of the experiment (8 parts FCS and 2 parts dimethylsulfoxide). Cells were distributed into aliquots with a number of 2x10^6^ cells per vial in a 1:1 dilution of cell culture medium and cryo-medium (chilled to 4 °C). Vials were placed in a precooled (−20 °C) cryo-vial container over-night, and then stored at −80 °C. The recovery rate directly after thawing was between 40 and 50 %. Total cell numbers were counted with a “Neubauer-improved” counting chamber before freezing and after thawing (Carl-Roth, Karlsruhe, Germany). The counted mean values of all experimental repetitions before freezing were defined as 100 %. The recovery rate was calculated due to setting the counted mean values of all experimental repetitions after re-thawing in relation to the 100 % before freezing.

### Microelectrode array recordings (MEA)

We used MEAs with a square grid of 60 Ti/TiN electrodes (30 μm diameter, 200 μm spacing) and an input impedance of <50 kΩ (manufacturer: Multi Channel Systems, Reutlingen, Germany). The measurements were performed with the standard software MC_Rack by Multi Channels Systems. The details of the technical and statistical principles adopted have been already described elsewhere (Illes et al. 2009). Briefly, the number of action potentials (spikes) and burst events per minute was aggregated across all active electrodes. Single channel burst events occurred when a neuron fired >3 spikes in a short time, preceded and followed by a quiescent period. Inter-burst intervals were calculated as time between two successive bursts, and burst duration as interval between first and last spike in a burst. Spike synchrony was assessed by calculating the chance exceeding coincidence ratio of spikes binned in 10 ms intervals. Cohen’s kappa reflects firing synchrony by averaging this ratio across all active electrode pairs. A small kappa value close to zero denotes asynchronous spiking, while a kappa of one is obtained for complete synchrony.

### Immunocytochemistry

For immunocytochemistry, cells were seeded on poly-d-lysine- and laminin-coated cover slips (Coverslips, VWR International, Darmstadt, Germany; recombinant proteins Biopur, Reinach, Switzerland). After 3 days under the influence of bFGF (20 ng/ml, Tebu-bio, Offenbach, Germany) the cells were fixed with 4 % PFA (Roti-Histofix, Carl Roth, Karlsruhe, Germany) for 15 min at room temperature. Cells were blocked for 30 min at room temperature with onefold Roti-Immuno-Block containing 0.25 % Triton X-100 for permeabilisation (Carl-Roth, Karlsruhe, Germany) and incubated with the following primary antibodies at 4 °C overnight anti-βIII-tubuline (Tuj1; 1:500; R&D Systems, Minneapolis, USA), anti-glial fibrillaric acid protein (GFAP) (1:500; Dako, Hamburg, Germany), anti-vesicular GABA transporter (VGAT) (20 μg/ml; 1:250; Millipore, Billerica, MA, USA), anti-vesicular glutamate transporter 1 (VGLUT1) (1 μg/ml; 1:1,000; Millipore, Billerica, MA, USA) antibody. For detection of primary antibodies, fluoresceine-isothiocyanate- (FITC; 1:500; Millipore, Billerica, MA, USA) or indocarbocyanine—(Cy3; 1:800; or Cy5; 1:200; Millipore, Billerica, MA, USA) coupled secondary antibodies were used. The first and secondary antibodies were diluted in onefold Roti-Immuno-Block without Triton X-100 (Carl Roth, Karlsruhe, Germany). For visualization of cell nuclei, cells were co-stained with DAPI (Invitrogen, Karlsruhe, Germany). For negative controls, primary antibodies were omitted in each experiment.

### CTB-assay

CellTiter-Blue (CTB) cell viability assay was performed according to the manufacturer’s guidelines (Promega, Madison, WI, USA). CTB was added to the cells into the culture medium in a 1:5 dilution and incubated for 4 h at 37 °C in a humidified atmosphere. CTB/medium mix was removed from the cells and measured in a spectrophotometer (excitation: 560 nm, emission: 590 nm). Living cells are able to convert resazurin to the fluorescent form resorufin in their mitochondria under NADH^+^ usage, which is measureable due to its fluorescence. Increasing CTB values during the experiments were declared as high metabolic activity of the cultured cells.

### CytoTox-ONE assay (LDH-assay)

CytoTox-ONE assay was performed according to the manufacturer’s guidelines (Promega, Madison, WI, USA). 100 μl cell culture medium was removed from the cells and substrate was added to the cell culture medium in 1:1 dilution and incubated for 30 min at room temperature in a dark chamber. The mix was measured in a spectrophotometer (excitation: 560 nm, emission: 590 nm). Under the influence of the assays substrate, resazurin is converted to the fluorescent form resorufin due to the lactate dehydrogenase (LDH) which is released to the medium by dead cells only. Therefore, increasing values during the experiments were declared as increasing cell death.

### Real-time quantitative RT-PCR

RNeasy Kit (Qiagen, Hilden, Germany) was used for RNA isolation of cultured E18 rat cortical cells. Then, a reverse transcription into cDNA (ABI, Darmstadt, Germany) was performed. Real-time quantitative PCR was carried out by the usage of the 7,500 fast or 7,500 real-time quantitative PCR cycler (ABI, Darmstadt, Germany). SYBR green master mix (Qiagen, Hilden, Germany) or equivalent chemistry from another supplier (Quantace, London, UK) was used. The specific primers for genes of interest and the housekeeping gene GADPH (glyceraldehyde-3-phosphate dehydrogenase,) were purchased from Qiagen (QuantiTect primer assays, Qiagen, GAPDH set #QT00199633, GFAP set #QT00195517, βIII-tubuline set #QT00188819). The genes of interest (target gene) in the freshly prepared group or cryo-preserved cells group (PBS-treated) were analyzed in at least 3 independent cultures in triplicate each. Every experiment in the “fresh” or the “frozen” group provided delta C_T_ values (ΔC_T_: gene of interest minus reference gene), where the threshold cycle for the housekeeping gene was subtracted from the threshold cycle of the gene of interest. Cells from 3 different independent experiments were harvested for mRNA extraction, the presented values are mean ± standard error of mean (SEM).

### Statistical analyses

Experiments were repeated with independent cultures at least three times in triplicate each. The resulting data sets were statistically analyzed and illustrated using the GraphPad Prism 4 (GraphPad Software Inc., San Diego, CA, USA, 2003) software. For approval of statistical significance between groups, a two-tailed unpaired *t* test was performed. *P* values < 0.05 were considered to indicate significant differences.

## Results

### Immunocytochemical characterization of fresh and frozen primary rat cortical cells

**Fig. 1:**
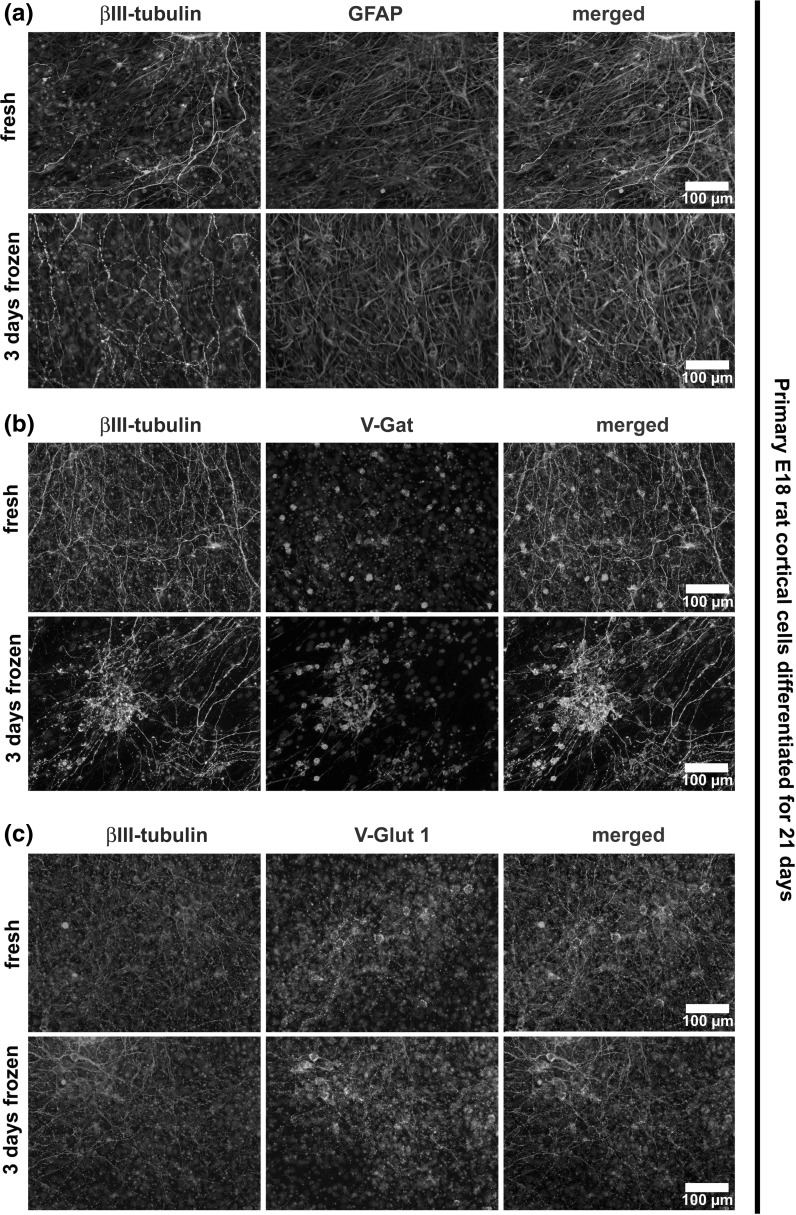
**a** Immunocytochemical stainings showed similar expression of the neuronal marker βIII-tubulin and the astrocytic marker GFAP in both experimental groups (freshly seeded vs. thawed cells) after 21 days in culture. Thawed cells had been frozen at −80 °C for 3 days. **b** Both experimental groups (freshly seeded vs. thawed cells after 21 days in culture) showed similar ratios of GABAergic neurons, as indicated by co-stainings against βIII-tubulin and the vesicular GABA transporter VGAT. Thawed cells had been frozen at -80 °C for 3 days. **c** Similar amounts of glutamatergic synapses were observed in both experimental groups (freshly seeded vs. thawed cells after 21 days in culture) by staining against the vesicular glutamate transporter 1 (VGLUT1). Thawed cells had been frozen at -80 °C for 3 days

E18 rat cortical cells were characterized in two parallel experimental groups: they were either (a) directly seeded or (b) first frozen for 3 days, thawed and then seeded (for experimental paradigm and procedure of cryopreservation and thawing see “Materials and methods” section). After cultivation for 21 days, a dense layer of βIII-tubulin-positive neurons and GFAP-positive astrocytes had grown in both experimental groups, and no gross differences were seen by visual inspection (Fig. 1a).

We also investigated the amount of inhibitory (vesicular GABA transporter: VGAT) and excitatory (vesicular glutamate transporter 1: VGLUT1) synapses in cultures of both fresh and frozen cell populations by means of immunocytochemistry (Fig. 1b and 1c). For this purpose, we performed a co-staining against βIII-tubulin to mark all neurons and against the indicated vesicular neurotransmitter transporter. We could not detect any differences in both experimental groups by visual inspection.

### Gene-expression of neuronal and glial markers and vitality and viability in fresh and frozen primary rat cortical cells

**Fig. 2:**
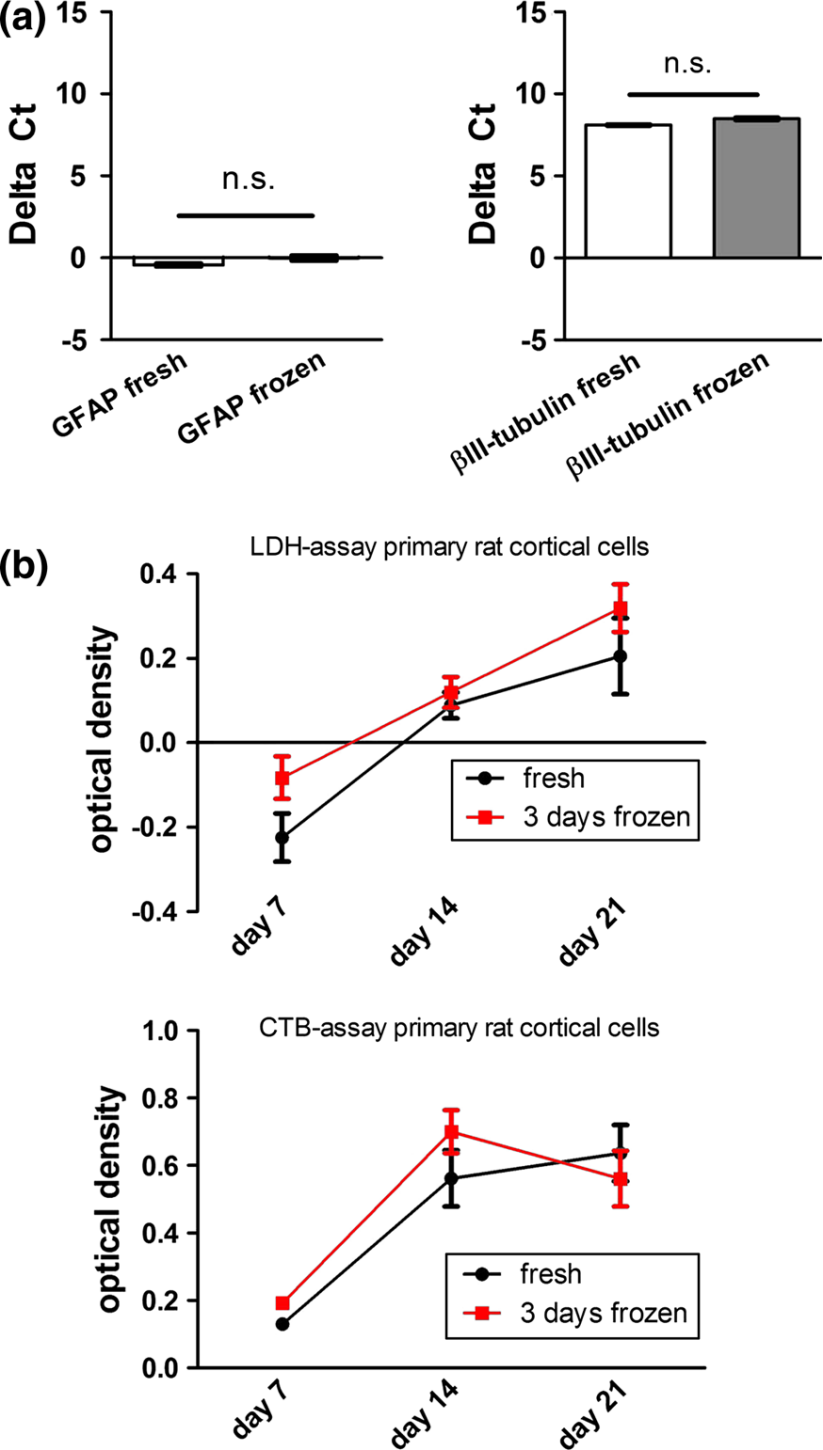
**a** Results from quantitative real-time PCRs are shown: Gene-expression levels of βIII-tubulin and GFAP were similar in the two experimental groups after 21 days in culture. **b** Both experimental groups exhibited similar viability and vitality at 7, 14 or 21 days in vitro as assessed by LDH and CTB assays

For a more detailed characterization, we investigated the expression levels of βIII-tubulin- and GFAP (glial fibrillary acidic protein)-mRNAs as indicators for neurons and astrocytes. We calculated delta C_T_ as the difference of threshold cycle with respect to a housekeeping gene GADPH and found no significant differences in delta C_T_ between the freshly prepared and cryo-preserved (3 days) group (Fig. 2a) after 21 days in culture. Cells from 3 different independent experiments were harvested for mRNA extraction, the presented values are mean ± SEM. Moreover, we compared the vitality and the viability of fresh and frozen cells by means of CytoTox-ONE and CTB assay. The CytoTox-ONE assay measures the relative amount of LDH in the medium which is released by dead cells only. Therefore, increasing values during the experiments represented increasing cell death (reduced cell viability). The CTB assay measures the metabolic activity of living cells. Increasing CTB values during the experiments were regarded as a sign of high metabolic activity of the cultured cells (high vitality). For experiments, the cells were seeded onto coverslips, and measurements were performed at day 7, 14 and 21 in culture. We were not able to detect significant differences in the viability and vitality between the frozen and the fresh group (Fig. 2b).

### In-vitro neuronal network activity of fresh and frozen primary rat cortical cells

**Fig. 3:**
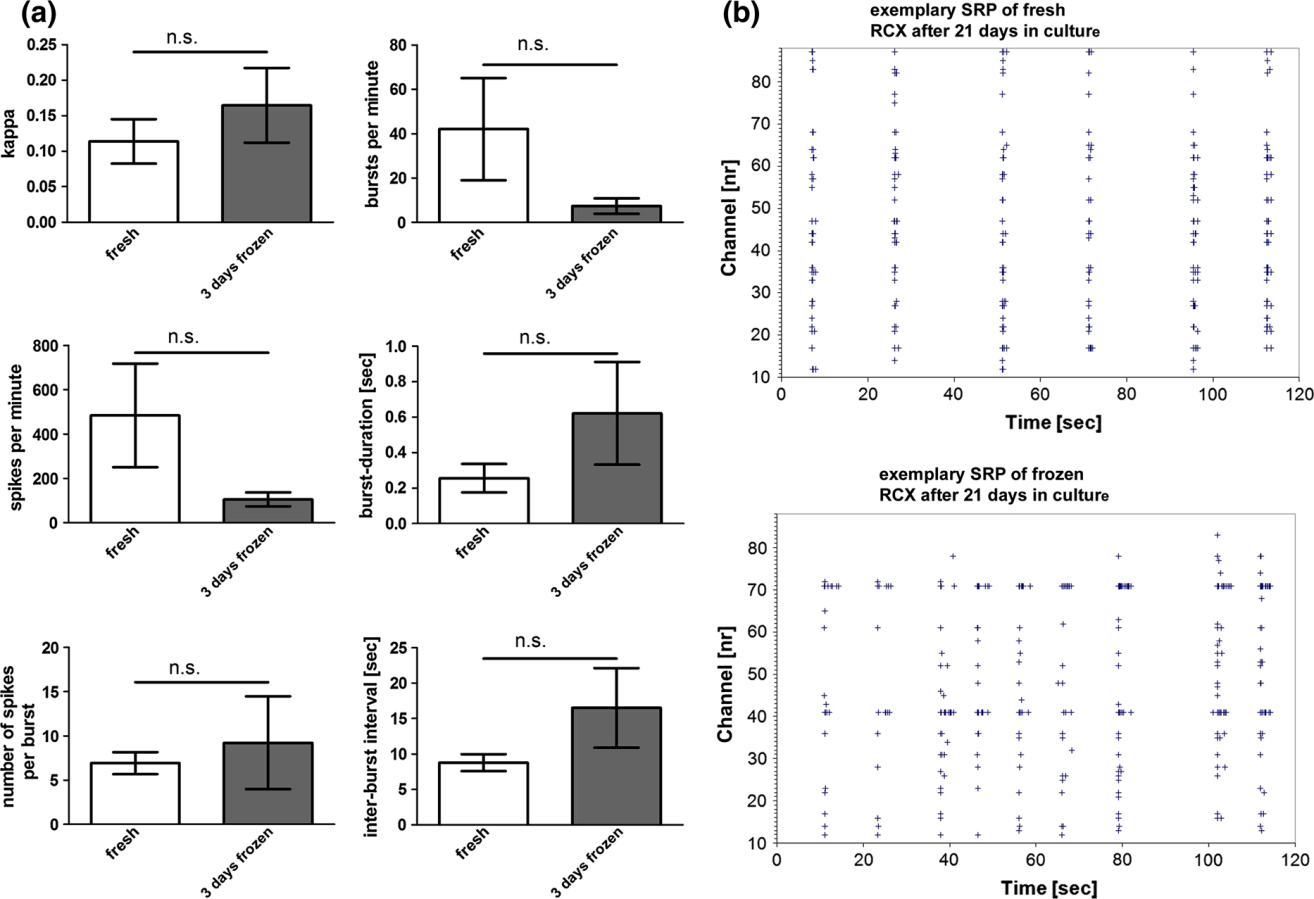
**a** Both experimental groups exhibited similar electrical activity on microelectrode arrays after 21 days in culture. Activity was characterized by the six parameters Cohen’s kappa, number of bursts per minute aggregated across all electrodes, number of spikes per minute aggregated across all electrodes, burst duration, number of spikes per burst and inter-burst interval. **b** Both experimental groups exhibited similar spatiotemporal firing patterns on microelectrode arrays. Exemplary SRPs represent 120 s of MEA recordings. Different MEA electrodes are plotted on the vertical axes and time is plotted on the horizontal axis. Each “+” represents a single action potential. *Vertical rows* of “+” indicate synchronous population burst activity of the entire network

We investigated in vitro neuronal network activity of fresh and frozen/re-thawed cells that were cultured for 21 days. Analyzed parameters were global activity markers like spike or burst rates, burst duration and inter-burst interval and parameters that measure the degree of network synchrony like Cohen’s kappa. We were not able to detect significant differences between the groups (Fig. 3a). Exemplary spike raster plots (SRPs) of both groups showed similar synchronous burst activity (Fig. 3b). The values in this figure represent 3 independent preparations (cultures). The data of 3 different MEA chip recordings were used (values are mean ± SEM).

## Discussion

In mammalian cell culture, cryopreservation is used as a standard method to store and bank different cell types (Grout et al. 1990; Morris 2007). Plenty of these cells are more or less robust tumor cell lines with mutations in genes that code for regulating cell cycle proteins (Odell et al. 2010) or cells immortalized by genetic manipulation with oncogenes (for instance c-myc) (Drayton and Peters 2002; Gonos and Spandidos 1993; Stacey and MacDonald 2001). The post-thaw vitality of cryo-preserved cells often depends on slow cooling rates, fast defrosting, liquid nitrogen long-term-storage and the usage of cryo-protectants (Grout et al. 1990; Morris 2007). Different theories are dealing with the physical backgrounds of the so called “cryo-sickness”. The most prominent ones focus on osmotic stress formation due to extra- and intracellular ice formation followed by dehydration and cell shrinking (Muldrew and McGann 1990, 1994). Various studies in the past were aimed at optimizing the freezing conditions for primary dissected brain tissue, since the post-thaw vitalities of this sensitive tissue are often very low (Paynter 2008). The freezing of whole blocks of embryonic rat cortices, the interval freezing or more sensitive methods for tissue homogenization are only some of the described approaches (Das et al. 1983; Fang and Zhang 1992; Jensen et al. 1987). To our knowledge, only one study demonstrated that neuronal cells are electrically active after the thawing process: Otto et al. (2003) examined commercially available cryo-preserved neurons on MEAs and found them comparable to cultures from freshly dissociated cells. We show here that it is possible to cryo-preserve and re-thaw freshly prepared E18 rat cortical cells with an easy and reliable protocol at least for a short period. In our experiments, these cells generated synchronous neuronal network activity on MEA chips after a 3-week cell culture period. We found no significant differences between frozen/thawn or freshly dissected cells regarding global activity or network synchrony. Henceforth, despite a freeze and thaw cycle neural populations were able to form multiple functional synapses leading to mature networks exhibiting population bursting. We found no differences in the composition of the mature cell populations that were formed after a cultivation period of 21 days. We analyzed this by means of immunocytochemistry against βIII-tubulin (neurons) and GFAP (astrocytes), as well as against VGAT (inhibitory neurons) and VGLUT1 (excitatory neurons). We also analyzed the gene expression of βIII-tubulin and GFAP at mRNA level after differentiation and found no significant differences between the fresh and the cryo-preserved group. We further found no differences in the viability or the vitality of the fresh and frozen cells at different time points of their maturation. Future experiments should be aimed at enlarging the frozen storage time of primary brain cells, to a long-term maximum up to several years. The experimental paradigm used in this study is based on the theory that the phase transition from liquid to frozen cryo-medium is the critical step in cryopreservation. Therefore it may not be important how long the cells are stored but how well they can survive the phase transition between frozen and liquid cryo-medium. Nevertheless a proven long-time storage in the frozen state would make the new method even more attractive. Taken together, our findings will enable experiments with neuronal cell cultures that are independent from timed animal pregnancy, animal breeding or expensive commercially available cells. In this way, time and cost for experiments can be kept low and the overall amount of sacrificed animals can be drastically reduced in accordance to 3R (replace, reduce, refine) approaches.
